# Prevention of dementia: a necessary worldwide agenda

**DOI:** 10.1002/alz70860_095994

**Published:** 2025-12-23

**Authors:** Gill Livingston, Lancet commission on dementia prevention, intervention and care

**Affiliations:** ^1^ Psychiatry, University College London, London, London, United Kingdom, North London NHS Foundation trust, London, London, United Kingdom

## Abstract

There has been a hugely welcome reduction in risk for dementia in some High‐Income Countries. However, this has been restricted to those of higher income and more educated people. The 2024 Lancet commission found 14 risk factors with enough evidence to include them as potentially modifiable risks with a 45% theoretical potential reduction worldwide dementia. I will discuss the evidence for risks and further evidence since the commission and that tackling these risks makes a difference to and whether and how these actions can be afforded.

